# Cell Properties of Lung Tissue-Resident Macrophages Propagated by Co-Culture with Lung Fibroblastic Cells from C57BL/6 and BALB/c Mice

**DOI:** 10.3390/biomedicines9091241

**Published:** 2021-09-16

**Authors:** Mayu Tsurutani, Haruka Horie, Kazushige Ogawa

**Affiliations:** 1 Laboratory of Veterinary Anatomy, Graduate School of Life and Environmental Sciences, Osaka Prefecture University, 1-58 Rinku-Ourai-Kita, Izumisano, Osaka 598-8531, Japan; swc01031@edu.osakafu-u.ac.jp; 2 Laboratory of Veterinary Anatomy, College of Life, Environment and Advanced Sciences, Osaka Prefecture University, 1-58 Rinku-Ourai-Kita, Izumisano, Osaka 598-8531, Japan; sac01031@edu.osakafu-u.ac.jp

**Keywords:** lung interstitial macrophages, alveolar macrophages, co-culture, BALB/c mice, C57BL/6 mice

## Abstract

Tissue-resident macrophages (Mø) originating from foetal precursors are maintained by self-renewal under tissue/organ-specific microenvironments (niches). We recently developed a simple propagation method applicable to tissue-resident Mø by co-culturing. Here, we examined the properties of lung tissue-resident Mø propagated by co-culturing with lung interstitial cells. The intracardially and intratracheally perfused lung from BALB/c and C57BL/6 mice could minimise the contamination of alveolar Mø and lung monocytes. Lung tissue-resident Mø could be largely propagated under standard culture media along with the propagation of lung interstitial cells demonstrating a fibroblastic morphology. Propagated lung Mø showed characteristic expression properties for Mø/monocyte markers: high expressions of CD11b, CD64 and CD206; substantial expressions of Mertk; and negative expressions of Ly6C, MHC II and Siglec-F. These properties fit with those of lung interstitial Mø of a certain population that can undergo self-renewal. Propagated fibroblastic cells by co-culturing with lung Mø possessed niche properties such as *Csf1* and *Tgfb1* expression. Propagated lung Mø from both the mouse types were polarised to an M2 phenotype highly expressing arginase 1 without M2 inducer treatment, whereas the M1 inducers significantly increased the iNOS-positive cell percentages in C57BL/6 mice relative to those in BALB/c mice. This is the first study to demonstrate fundamental properties of lung tissue-resident Mø propagated by co-culturing. Propagated lung Mø showing features of lung interstitial Mø can serve as an indispensable tool for investigating SARS-CoV-2 diseases, although lung interstitial Mø have gained little attention in terms of their involvement in SARS-CoV-2 disease pathology, in contrast to alveolar and recruited Mø.

## 1. Introduction

Macrophages (Mø) are multifunctional cells that are indispensable for the development and regeneration of tissues/organs. They also assist in the removal of pathogens invading the body. Mø reside in various tissues/organs as heterogeneous populations demonstrating tissue/organ-specific functions. Majorly, two types of Mø function in adults: (1) tissue-resident Mø, which colonise tissues/organs at a steady state and perform tissue/organ-specific functions to maintain the tissue/organ homeostasis, and (2) recruited Mø, which originate from bone marrow-derived monocytes that circulate in the blood and infiltrate lesions in response to damage of the tissues/organs. Previously, all tissue-resident Mø in adults were considered to originate from the bone marrow-derived monocytes that undergo tissue/organ-specific differentiation. However, based on the recently accumulated evidence, majority of the tissue-resident Mø in adults are believed to originate from foetal Mø in the yolk sac and/or foetal monocytes in the foetal liver. These foetal precursors migrate to diverse tissues/organs during embryonic development, colonise in tissue/organ-specific microenvironments (niches), and then undergo tissue/organ-specific differentiation into tissue-resident Mø as well as persist into adulthood through self-maintenance of the local proliferation processes in a steady state, independent of any input from bone marrow-derived monocytes [1,2,3]. In contrast, it has been reaffirmed that tissue-resident Mø that colonise the interstitial tissues of several tissues/organs such as intestines and dermis are terminally differentiated cells that do not proliferate locally in the colonising tissues in a steady state and are gradually replaced by blood monocyte-derived Mø [4,5]. Thus, we speculated that tissue-resident Mø originating from foetal precursors can proliferate in vitro under suitable conditions. Guilliams et al. recently proposed that tissue-resident Mø that receive nourishment under suitable niches possess self-renewal properties and can undergo tissue/organ-specific differentiation [6,7]. Therefore, we hypothesised that tissue-resident Mø that originated from foetal precursors in a certain organ could be propagated alongside the propagation of niche-forming cells residing in the respective organ. We then accordingly and successfully developed a simple propagation method of tissue-resident Mø by co-culturing with the respective tissue/organ-residing cells. This method can be commonly applied to tissue-resident Mø such as the brain, liver, spleen and lung [8]. Mø from bone-marrow-derived monocytes and monocyte/Mø cell lines have been widely applied for in vitro studies on Mø in general. Ex vivo methods have also been primarily applied for the study of tissue-resident Mø. Thus, the co-culture method has opened possibilities to use several types of tissue-resident Mø for diverse research applications in vitro similar to those for the currently used adult monocyte-derived Mø.

Two types of tissue-resident Mø—alveolar Mø and lung interstitial Mø—can colonise in the lung. Alveolar Mø are well-defined and representative Mø that originate from foetal monocytes in the foetal liver, which populate the alveolar and airway lumen, are nursed by alveolar epithelial cells as the niche, and play important functions in surfactant homeostasis, pathogen clearance and immune homeostasis [9,10,11]. On the other hand, lung interstitial Mø are non-alveolar Mø that populate the lung interstitium such as the bronchial and alveolar interstitium. They were not well-defined owing to the technical challenges encountered during cell preparation and fallacious conception as being intermediate cells in the development of AMø, albeit they have recently gained attention owing to their immunoregulatory functions [12,13]. It is thus evident that lung interstitial Mø in adult mice are composed of two distinct subpopulations: cells originating from foetal Mø in the yolk sac and cells from bone-marrow blood monocytes [14]; and cells located in the bronchial interstitium that demonstrate self-renewal property and cells located in the alveolar interstitium that are gradually replaced by blood monocyte-derived Mø [15]. It is believed that tissue-resident Mø that receive nourishment under suitable conditions possess self-renewal properties and undergo tissue/organ-specific differentiation [6,7]. Based on this, we hypothesised that lung interstitial Mø originating from foetal Mø may be propagated alongside niche-forming cells residing in the lung interstitium. We also speculated that the propagated Mø exhibit similar expression patterns of Mø markers to those in ex vivo lung interstitial Mø of foetal origin because of their propagation with niche-forming cells. We successfully propagated tissue-resident Mø from mouse lung using the co-culture method that we developed previously [8]. Lung tissue-resident Mø were propagated along with the fibroblastic cells among diverse lung cells. To our knowledge, there are few studies describing the in vitro propagation of lung interstitial Mø and their M1 (classically activated) and M2 (alternatively activated) polarisation properties in vitro. Thus, we examined the expression of Mø markers and M1/M2 polarisation properties in propagated lung Mø as well as properties of fibroblastic cells as the niche.

Severe acute respiratory syndrome−coronavirus 2 (SARS-CoV-2) infection leads to the development of a hyperinflammatory syndrome that has been deemed the major cause of death as well as in bilateral interstitial pneumonia, which often leads to acute respiratory distress syndrome and pulmonary fibrosis among survivors of the coronavirus disease 2019 (COVID-19) [16,17]. It has thus become apparent that that dysregulated responses of alveolar Mø and the recruited Mø are deeply involved in the pathology of the hyperinflammatory syndrome and pulmonary fibrosis [16,18]. Therefore, we believe that propagated tissue-resident Mø from the lung can be a highly useful tool to evaluate the pathology of COVID-19 in vitro as well as in the development of new drugs against COVID-19. From this perspective, we attempted to reveal the basic properties of propagated lung tissue-resident Mø by examining the properties of Mø and by comparing them with those of the alveolar Mø. It has been reported that blood monocyte-derived Mø from BALB/c and C57BL/6 strain mice are more easily polarised to M2 and M1 phenotypes, respectively [19,20]. In this regard, we also examined the M1/M2 polarisation properties of propagated tissue-resident Mø from the lungs of BALB/c and C57BL/6 mice.

## 2. Materials and Methods

### 2.1. Animals

Specific-pathogen-free BALB/c and C57BL/6 male mice were obtained from Japan SLC, Inc. (Hamamatsu, Japan). The animals had been maintained under the standard housing condition in a clean-grade environment on a 12-h light-dark cycle and fed with a standard diet and *ad libitum* water. In total, 27 BALB/c and 27 C57BL/6 mice of age 7–8 weeks were used in this study. The animal experimentation protocol was approved by the Animal Research Committee of the Osaka Prefecture University (approval number: 19-49, 20-32, 21-26). All experiments were performed following the relevant guidelines and regulations of the Osaka Prefecture University.

### 2.2. Collection of Alveolar Mø

Mice were sacrificed by injecting an overdose of pentobarbital intraperitoneally (150 mg/kg body weight; Somnopentyl, SOMO4-YA1706, Kyoritsu Seiyaku, Tokyo, Japan), followed by intracardial perfusion with Ca/Mg-free Hanks′ Balanced Salt Solution (HBSS; H6648, Sigma-Aldrich, St Louis, MO, USA) supplemented with 50 U/mL heparin (224122485, Mochida Pharmaceutical, Tokyo, Japan) to remove the blood. The lung was aseptically dissected and immediately dipped in ice-cold HBSS. Next, the adipose tissues surrounding the pulmonary hilum from the lung were removed. To collect the alveolar Mø, the bronchoalveolar lavage fluid from three mice was used as one sample. A 21-gauge intravenous catheter was inserted into the trachea, and approximately 4 mL of HBSS was injected and then immediately withdrawn a few times. The bronchoalveolar lavage HBSS fluid was then sedimented at 100× *g* for 5 min, after which the alveolar cells were plated in a 5.5 cm bacteriological Petri dish (1-8549-02; As One, Osaka, Japan) containing DMEM (D6046, Sigma-Aldrich, St Louis, MO, USA) and supplemented with 10% foetal bovine serum (FBS; 175012, Nichirei Biosciences Inc., Tokyo, Japan), 100 U/mL penicillin and 100 μg/mL streptomycin (pen/strep; P4333, Sigma-Aldrich, St Louis, MO, USA) (DMEM-FBS). The adherent cells on the dish were regarded as alveolar Mø and used for RT-PCR analyses and flow cytometry analyses.

### 2.3. Propagation of Tissue-Resident Mø by Co-Culturing with Interstitial Cells Obtained from the Lung

The lung tissue-resident Mø were cultured and propagated according to the method described by Ogawa et al. with some modifications [8]. Briefly, after clearing the alveolar cells through bronchoalveolar lavage, the entire lung was minced with a razor blade and transferred to 15-mL conical tubes containing 8 mL cell dispersion enzyme solution: 20 mM Hepes-buffered HBSS (pH 7.4) including 0.5 mg/mL Collagenase Type IA (C9891, Sigma-Aldrich, St. Louis, MO, USA) and 1 mM CaCl_2_. These tissues were then digested at 37 °C for 50–60 min with gentle stirring at 120 rpm, with one change in the digestion solution. After washing with HBSS, the cell/tissue suspensions were further dispersed by pipetting. The suspensions were sedimented at 100× *g* for 5 min (Model 2410, Kubota, Tokyo, Japan). The lung cells/tissues per mouse were plated on three 10-cm tissue-culture dishes (3020-100, AGC Techno Glass, Haibara, Japan) and then cultured in DMEM-FBS. The medium was refreshed every 3–4 days until the dishes were covered with multi-layered cells composed of Mø and other lung interstitial cells such as fibroblasts. Over-confluent cells were then detached by 0.1% trypsin/2 mM EDTA in HBSS, followed by pipetting. Subsequently, the cells at a dilution ratio of 1:3 were subcultured or frozen at −80 °C in a cell suspension with Bambanker (CS-02-001, Nippon Genetics, Tokyo) as a cryopreservative and then maintained in the same medium until they gained over-confluence again.

### 2.4. Separation of Lung Tissue-Resident Mø Propagated by Co-Culture from Interstitial Cells

Co-cultured lung tissue-resident Mø were separated from lung interstitial cells according to the method by Ogawa et al. [8]. Briefly, co-cultured, over-confluent cells up to four passages (usually 1−2 passages) were used for the separation of Mø. The over-confluent cells harvested from a 10-cm tissue-culture dish were seeded in a 5.5 cm or 10 cm bacteriological Petri dish (1-7484-01, As One) containing DMEM-FBS. After several hours to 1 day, when the Mø selectively adhered onto the dish surface and interstitial cells usually formed nonadherent cell aggregates in the dish, the adherent cells were washed with conditioned media to remove nonadherent cells and used for RT-PCR analysis. For phagocytosis analysis and flow cytometry, the adherent cells were detached with 5-mM EDTA in 10 mM HEPES-buffered HBSS (EDTA-HEPES-HBSS), followed by pipetting. The cell suspension was passed through a cell strainer (352235, BD Falcon, Franklin Lakes, NJ, USA) to remove the cell aggregates, and then sedimented at 220× *g* for 5 min, followed by suspension in Ca/Mg-free phosphate-buffered saline (PBS; 1102P10, Cell Science & Technology Institute, Inc., Yamagata, Japan) containing 1% bovine serum albumin (BSA; A3059, Sigma-Aldrich, St. Louis, MO, USA), 2 mM EDTA and 0.01% NaN_3_ (BSA/EDTA-PBS), after which the number of cells was calculated and used for the further experiments.

Lung interstitial cells other than lung tissue-resident Mø were purified from nonadherent cells in the bacteriological Petri dish because Mø were included in the nonadherent cell aggregates as a minor population. The suspensions of the culture supernatant in the bacteriological Petri dish including nonadherent cells/aggregates were sedimented at 100× *g* for 5 min, incubated in 0.1% trypsin/2 mM EDTA in HBSS at 37 °C for approximately 5 min, and then sedimented again. Then, the cells were plated and cultured in a 10-cm tissue-culture dish containing DMEM-FBS. On the next day after plating, the cells were treated with 0.05% trypsin/1 mM EDTA in HBSS at 37 °C for approximately 3 min, when the interstitial cells, except for Mø were largely detached from the dish and most Mø remained adhered onto the dish. Then, the detached cells were collected, sedimented and cultured again in the tissue-culture dish containing DMEM-FBS. We repeated this experiment at least once more to purify the lung interstitial cells by removing the relatively strong-adhesive Mø for use in RT-PCR analysis.

### 2.5. Phagocytosis Analysis with Fluorescent Beads

We analysed the phagocytotic property in lung tissue-resident Mø propagated by co-culturing according to the method described by Ogawa et al. [8]. Briefly, after the separation on the bacteriological Petri dish, the cells (2.5 × 105/0.5 mL DMEM-FBS) were placed in a 5 mL tube that was siliconised (Siliconise L-25, 0411002, Fuji-Rika Industries, Osaka, Japan) according to the manufacturer’s protocol to prevent adhesion to the tube wall. After the addition of 1.0 µL fluorescent yellow-green-conjugated latex beads (mean diameter, 1.0 µm; L4655, Sigma-Aldrich, St Louis, MO, USA), these cells were incubated at 37 °C for 2 h with gentle shaking at 18 rpm on a seesaw-type shaker (Wave SI slim; Taitec, Koshigaya, Japan), followed by washing thrice with HBSS and plating on a 3.5 cm glass-bottom dish (3910-035, AGC Techno Glass) with 1.5 mL DMEM-FBS for approximately 2 h until almost all the cells adhered to the surface. After fixation with 10% formalin (16061-00, Kanto Chemical, Tokyo, Japan) in PBS for >10 min, phase-contrast and green fluorescence images of the same fields were captured using a 10× and 20× objective lens (IX71; Olympus, Tokyo, Japan). Cells engulfing >2 latex beads were denoted as Mø. We counted >660 cells per sample, and the percent of Mø in each mouse was calculated from independent experiments (three mice and three experiments for the lung cells). Data are presented as mean values ± SD.

### 2.6. Total RNA Extraction and Semi-Quantitative RT-PCR Analysis

Total RNA was isolated from alveolar Mø, propagated lung tissue-resident Mø, and lung interstitial cells with the TRI Reagent (TR118, Molecular Research Centre, Inc., Cincinnati, OH, USA), and RT-PCR analysis was performed as described elsewhere [21]. Briefly, 1 µg of total RNA was transcribed into the first-strand cDNA using M-MLV reverse transcriptase, RNase H^−^ (316-08151, Nippon Gene, Toyama, Japan) and an oligo (dT)_18_ primer, according to the manufacturer’s instructions. The niche-dependent transcription factors providing resident tissue-specific identities have been revealed in representative tissue-resident Mø (BACH2, CEBPβ, PPARγ, alveolar Mø; DTX4, RUNX3, intestinal Mø; ID3, LXRα, SPIC, Kupffer cells; ID2, RUNX3, Langerhans cells; SALL1, SMAD2, SMAD3, microglia; CEBPβ, GATA6, peritoneal Mø; BACH1, SPIC, red pulp Mø) [22,23]. Therefore, we examined the mRNA expressions of these transcription factors as well as the lineage-determining transcription factor PU.1 in propagated tissue-resident Mø by co-culturing with lung interstitial cells as well as alveolar Mø, including in the bronchoalveolar lavage, to determine the properties of the propagated Mø. To examine the expression patterns of these molecules, 0.5 µL of the 25-µL reaction mixture was amplified with Taq DNA polymerase (TaKaRa Ex Taq HS, RR006A; TaKaRa Bio Inc., Otsu, Japan) using the reverse-transcribed cDNA as the template. The primer pairs and the thermal cycling conditions used for PCR amplification in this study are illustrated in Supplemental Tables S1 and S2. The RT reaction was omitted for negative controls. The PCR products were separated on 1.5% agarose gels and visualised by ethidium bromide staining. We also examined the mRNA expressions of CSF1, CSF2 and IL34 as growth factors of Mø as well as TGFβ1 in lung interstitial cells propagated by co-culturing with lung tissue-resident Mø as well as TGFβ1 and TGFβR2 in propagated lung Mø and alveolar Mø.

### 2.7. Flow Cytometry

Flow cytometry was performed to examine the expression of Mø/monocyte markers (CD11b, integrin αM subunit; CD11c, integrin αX subunit; CD64, Fc-γ receptor 1; CD68, scavenger receptor class D; CD86, B7-2; CD115, colony-stimulating factor 1 receptor; CD116, colony-stimulating factor 2 receptor; CD169, Siglec-1, Sialoadhesin; CD184, C-X-C chemokine receptor type 4; CD206, mannose receptor C-type 1; F4/80, EGF-like module-containing mucin-like hormone receptor-like 1; Ly6C, lymphocyte antigen 6C; Mertk, myeloid-epithelial-reproductive tyrosine kinase; MHC II, major histocompatibility complex class II; Siglec-F, sialic acid-binding immunoglobulin-like lectin F) in lung tissue-resident Mø propagated by co-culturing and segregating using bacteriological Petri dishes according to the method described by Ogawa et al., [8] with some modifications. Monoclonal antibodies used in the flow cytometry analyses are listed in Supplemental Table S3. The cells at a concentration of approximately 5 × 10^5^ cells/mL in BSA/EDTA-PBS were fixed in 10% formalin in BSA/EDTA-PBS for approximately 20 min at RT. After washing with BSA/EDTA-PBS, the cells were permeabilised in 0.2% saponin (30502-42, Nacalai Tesque, Kyoto, Japan) in BSA/EDTA-PBS for 5 min at RT. To avoid non-specific Fc-gamma receptor-mediated binding of fluorochrome-conjugated antibodies, the cell suspensions approximately 2.0 × 10^5^ cells/50 µL) were pre-treated with 0.5 µg of anti-mouse CD16/32 antibody for 10 min at RT. To the 50-µL cell suspension, we added 0.5 µg FITC-conjugated anti-CD11b, 0.25 µg APC-conjugated anti-CD11c, 0.15 µg APC-conjugated anti-CD64, 0.15 µg FITC-conjugated anti-CD68, 0.125 µg FITC-conjugated anti-CD86 antibody, 0.5 µg FITC-conjugated anti-CD115, 0.1 µg APC-conjugated anti-CD116, 0.15 µg APC-conjugated anti-CD169, 0.15 µg FITC-conjugated anti-CD184, 0.25 µg APC-conjugated anti-CD206, 0.5 µg APC-conjugated anti-F4/80, 0.25 µg APC-conjugated anti-MHC II, 0.15 µg FITC-conjugated anti-Ly6C, 0.15 µg APC-conjugated anti-Mertk, and 0.15 µg APC-conjugated anti-Siglec-F antibody, in accordance with the manufacturer’s instructions, followed by incubation for 10 min at RT. After washing, 20,000 cells were analysed for their expression characteristics by flow cytometry (S3 Cell Sorter; Bio-Rad Laboratories, Hercules, CA, USA). As controls, we used cell suspensions pre-treated with the anti-mouse CD16/32 antibody and then treated with the same fluorochrome-labelled isotype control antibody of the same amount as the test antibody. The expression of marker molecules was determined from >3 independent experiments in Mø propagated from the lung tissues derived from >3 mice.

To highlight the differences between the propagated tissue-resident Mø and alveolar Mø, we also examined the CD11b and Siglec-F expression in alveolar Mø from the bronchoalveolar lavage through flow cytometry because the expression levels of these molecules were shown to be different between ex vivo lung interstitial Mø and alveolar Mø [13]. After incubation with the antibodies and washing, 10,000 cells were analysed for their expression characteristics by flow cytometry (CytoFLEX S; Beckman Coulter, Brea, CA, USA).

### 2.8. M1 and M2 Polarisation by LPS Plus IFN-γ and IL-4

We compared the polarisation property of the propagated lung Mø between BALB/c and C57BL/6 mice based on the report that Mø derived from blood monocytes in BALB/c and C57BL/6 mice are more easily polarised to the M2 and M1 phenotype, respectively, by polarisation reagents [19,20]. The combination of LPS and IFN-γ was used as an inducer for M1 polarisation, while IL-4 was used as an inducer for M2 polarisation, as previously reported for monocyte-derived Mø [24,25] and propagated spleen tissue-resident Mø [8]. We examined the expressions of iNOS and arginase 1 as an M1 and M2 polarisation maker, respectively, in propagated lung tissue-resident Mø treated with the M1 and M2 polarisation inducers by flow cytometry, in accordance with the method of Ogawa et al. [8] albeit with some modifications. For instance, we separated the lung Mø from co-co-cultured over-confluent cells at a steady state when the cell densities were considered to be saturated in a dish under microscopy. The cells (3.2 × 10^5^) were plated on a 3.5-cm bacteriological Petri dish (1-8549-01, As One) with 2.5-mL DMEM-FBS. A few hours after seeding when almost all cells adequately adhered on the dish, 20 ng/mL lipopolysaccharide (LPS; L4391, Sigma-Aldrich, St. Louis, MO, USA) plus 50 ng/mL interferon-γ (IFN-γ; AF-315-05-100UG, PeproTech, Rocky Hill, NJ, USA), 20 ng/mL interleukin-4 (IL-4; 21-8041-U020, Tonbo), or vehicle (DMEM-FBS) were added to the dish. At 4 h and 24 h after the addition, the cells were detached with EDTA-HEPES-HBSS. Cell suspensions at the concentration of 1.5 × 10^5^ cells/50 µL were pre-treated with 0.5 µg of anti-mouse CD16/32 antibody and then incubated with a mixture of 0.15 µg of FITC-conjugated anti-iNOS and 0.01 µg of APC-conjugated anti-arginase 1, subsequently followed by incubation for 10 min at RT. After washing, 20,000 cells were analysed for their expression characteristics by flow cytometry (CytoFLEX S). iNOS-positive and -negative fractions appeared in the treatment with the M1 inducers, while no iNOS expression was detected in the cells of the control and M2-inducer treatment groups from both BALB/c and C57BL/6 mice. Thus, we measured the percentage and mean fluorescence intensities (MFI) of iNOS-positive cells normalised to those of the isotype controls only in the M1-inducer treatment at 4 h and 24 h in three independent experiments. In contrast, arginase 1 was highly expressed in all three groups from both BALB/c and C57BL/6 mice and the expression levels were similar between the two time points measured in each group. The MFI of arginase 1 normalised to those of the isotype controls in BALB/c mouse lung Mø in 6 groups (control, M1 and M2 groups at 4 h and at 24 h) as well as 3 groups (control, M1 and M2 groups without subdividing the two time points) were compared with those in C57BL/6 mouse lung Mø in three independent experiments. Statistical analyses were performed with statistical software incorporated into Microsoft Excel. The differences in the percentages and MFI of iNOS-positive cells at 4 h and 24 h in the M1 groups and the MFI of arginase 1 at 4 h and 24 h in the 3 groups, as well as in the 3 groups without subdividing the two time points between BALB/c and C57BL/6 mice lung Mø, were evaluated by a Student’s *t*-test. *p* < 0.05 was considered to indicate statistical significance. All values represent the means ± SD.

## 3. Results

### 3.1. Propagation Behaviour of Co-Cultured Lung Mø

**Figure 1:**
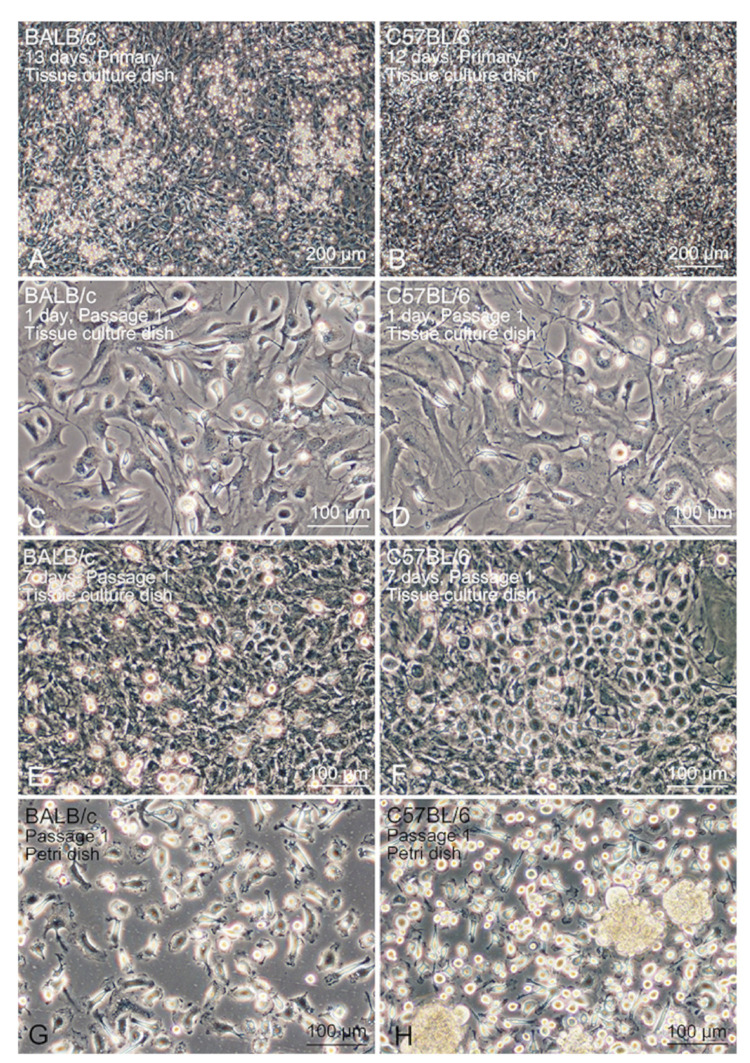
The propagation of lung tissue-resident Mø in co-culture and subculture with lung interstitial cells in tissue-culture dishes and their segregation on bacteriological Petri dishes. Right panels (**A**,**C**,**E**,**G**) and left panels (**B**,**D**,**F**,**H**) show the lung cells derived from BALB/c and C57BL/6 mice, respectively. (**A**,**B**): Primary lung cells cultured for the indicated days after seeding in a tissue-culture dish. (**C**,**D**): Lung cells after passage 1 conducted 1 day after seeding in a tissue-culture dish. (**E**,**F**): Lung cells after passage 1 conducted 7 days after seeding in a tissue-culture dish. (**G**,**H**): lung tissue-resident Mø in bacteriological Petri dishes. Mø selectively adhering to the dish surface and nonadherent cells forming cell aggregates in the (**H**) panel. Cell aggregates were removed by washing with a conditioned medium, as can be seen in the (**G**) panel.

Lung tissue-resident Mø were propagated by co-coculturing with interstitial cells from the mouse lung after clearing the alveolar cells through bronchoalveolar lavage. The propagation behaviours of the tissue-resident Mø by co-coculturing were similar between BALB/c and C57BL/6 mice: Mø showed high propagation in DMEM-FBS without any additional growth factors for Mø, such as CSF-1. By changing the culture media every 3–4 days, primary lung interstitial cells, including Mø, generally reached over-confluence within 2 weeks (Figure 1A,B). The over-confluent cells formed a multi-layered structure on a standard tissue-culture dish. The cells were then subcultured at a dilution ratio of 1:3 until reaching over-confluence again, which occurred within a similar period (Figure 1C–F). Alveolar epithelial cells were not engrafted/cultivated in the tissue-culture dish with the standard culture medium, because no clear cell masses that showed epithelial structures such as a cobblestone formation appeared in the co-culture after the first passage. Mø and interstitial cells in the co-culture were subcultured for more than four passages propagation and became over-confluent as observed for the co-cultured primary cells, and the over-confluent cells within 1–2 passages were usually used for expression analyses. The over-confluent co-cultured cells were frozen at a dilution ratio of 1:3 and then thawed, and the cultured frozen cells were treated to the same cultivating condition. The frozen cells were found to propagate similar to that by the unfrozen cells. Mø could be morphologically identified as small cells in the co-cultured lung interstitial cells, specifically, small flat cells with a few vacuoles, thin elongated cells with a few cytoplasmic protrusions, and small round or fusiform cells (Figure 1C,D).

### 3.2. Segregation of Mø by Adhesion to the Bacteriological Petri Dish

**Figure 2:**
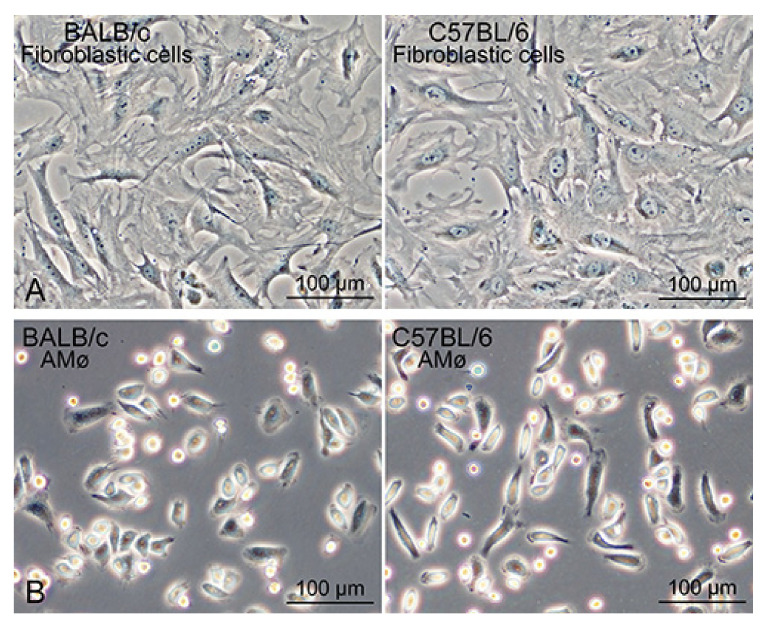

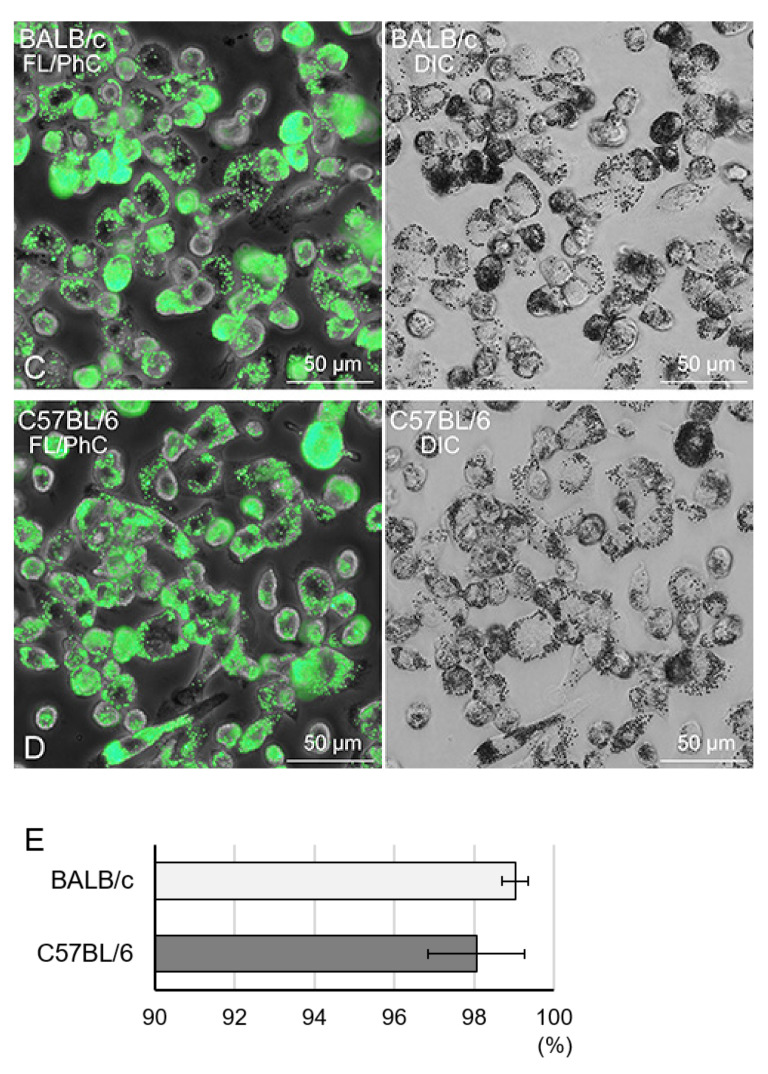
Phase-contrast images of lung fibroblastic cells and alveolar Mø from BALB/c and C57BL/6 mice as well as the propagated lung tissue-resident Mø demonstrating high phagocytotic activity. (**A**): Lung interstitial cells of BALB/c (**left** panel) and C57BL/6 mice (**right** panel) showing fibroblastic morphology. Lung interstitial cells propagated by co-culturing with lung tissue-resident Mø were separated based on the difference of adhesive property between the two cells. (**B**): Alveolar Mø of BALB/c (**left** panel) and C57BL/6 mice (**right** panel) in bacteriological Petri dishes. Alveolar Mø were separated from cells in the bronchoalveolar lavage fluids based on their selective adhesion to the dishes. (**C**–**E**): Lung tissue-resident Mø content (%) in cells segregated by adhesion to the bacteriological Petri dishes assessed by the phagocytosis of fluorescent beads. Cells adherent to the Petri dish were incubated with fluorescent beads of an average 1.0 µm diameter for 2 h and then fixed. Phase-contrast images (PhC), green fluorescence images (FL) and differential interference images (DIC) of the same fields were captured. More than 660 cells per sample were enumerated, and the percentages of Mø derived from three BALB/c and C57BL/6 mouse types each were determined from three independent experiments. Representative FL merged with PhC (**left** panels in (**C**,**D**)) and DIC of the same field (**right** panels in (**C**,**D**)) are shown. Bar graphs (**E**) showing the percent content of lung tissue-resident Mø, presented in mean ± SD (BALB/c, 99.0% ± 0.3%; C57BL/6, 98.1% ± 1.2%).

The propagated Mø were separated from the other lung interstitial cells in co-culture based on their different adhesive property to bacteriological Petri dishes, in which only the Mø adhered to the dish. Within a few hours of seeding co-cultured over-confluent cells, small round/fusiform cells with a few cytoplasmic protrusions, i.e., Mø adhered to the dish surface (Figure 1G,H), and cell aggregates floating in the media were also evident. These cell aggregates could be easily removed by washing with conditioned media. The cell density of Mø was almost unchanged, with or without cell aggregates, in the dishes when the culture continued for a few days. We usually collected more than 1.5 × 10^6^ adherent cells per 10-cm of bacteriological Petri dish and used them for analyses to determine the features of Mø. To examine the niche of lung Mø propagating in vitro, we collected lung interstitial cells other than Mø in the co-culture using a different detachment behaviour between the two cells treated with low-concentration trypsin for a short time in the tissue-culture dishes. The lung interstitial cells separated from Mø, which were easily detached by the treatment, mostly comprised large cells with a similar shape such as fibroblasts in the tissue-culture dishes (Figure 2A). Alveolar Mø with strong adhesion properties were also purified from the cells in the bronchoalveolar lavage by their adhesion onto bacteriological Petri dishes (Figure 2B) and then used for analyses to compare the features of propagating tissue-resident Mø with alveolar Mø.

Phagocytosis of fluorescent beads was evaluated to precisely determine the percentage of Mø in the collected segregated cells. Bead-phagocytotic features of the propagated tissue-resident Mø were found to be similar between BALB/c and C57BL/6 mice. During incubation, almost all cells were segregated from lung fibroblastic cells that phagocytosed the fluorescent beads (Figure 2C,D). Most of the cells contained numerous beads in their cytoplasm, and the cytoplasm of some cells was filled with beads. This observation demonstrated that the Mø propagated in co-culture possessed a high phagocytic property. The bead-positive and negative cells were enumerated to estimate the percentage of Mø in the segregated cells. We defined cells phagocytosing more than two beads as bead-positive cells and counted >660 cells per sample. Overall, these cells comprised 99.0% ± 0.3% Mø from BALB/c and 98.1% ± 1.2% Mø from C57BL/6 (Figure 2E). Thus, Mø segregation according to their property of adhesion to the bacteriological Petri dish represents a simple method to purify Mø from lung fibroblastic cells in co-culture.

### 3.3. Expression Profiles of Transcription Factors in Propagated Lung Tissue-Resident Mø

**Figure 3:**
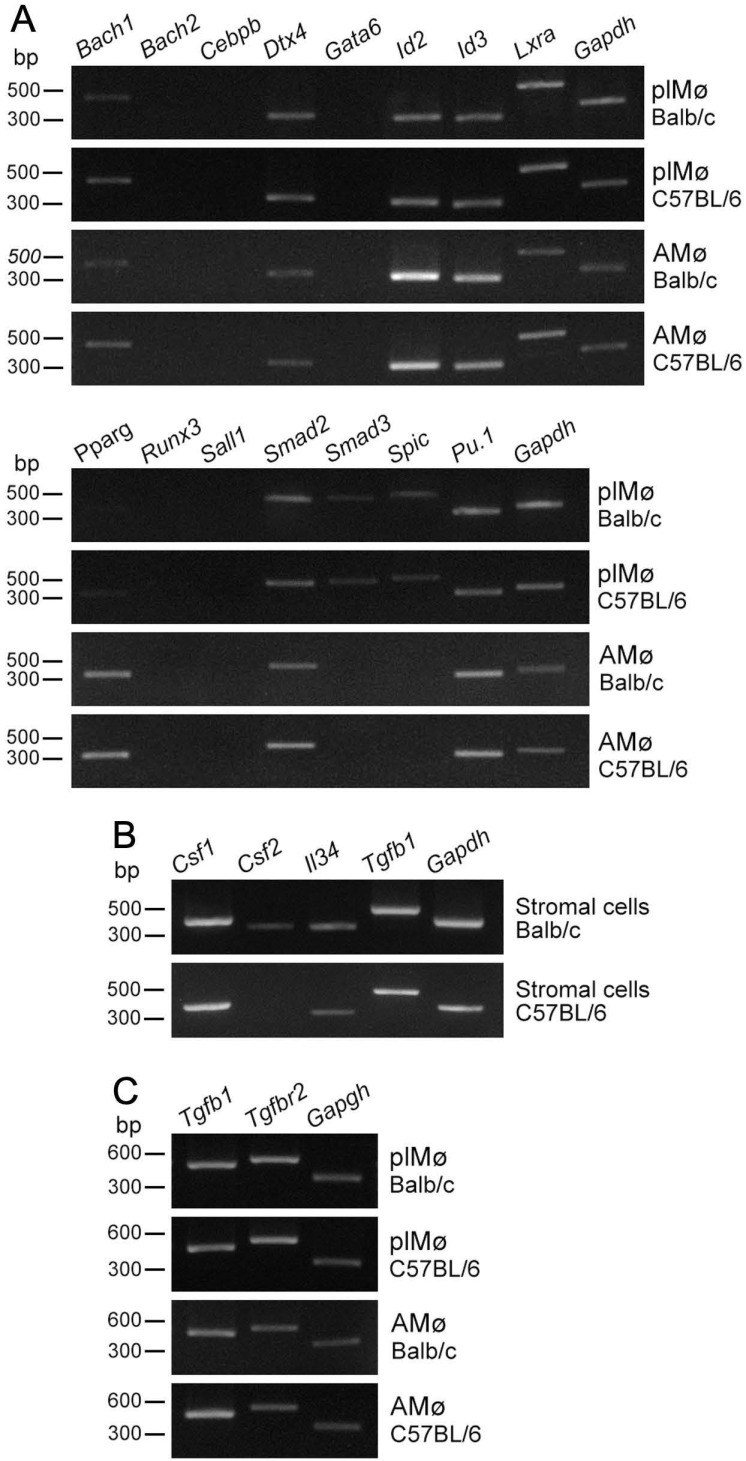
RT-PCR amplification of the transcription factors, *Tgfb1* and *Tgfbr2* in propagated lung tissue-resident Mø and alveolar Mø as well as that of the Mø growth factor mRNAs and *Tgfb1* in lung fibroblastic cells propagated by co-culturing in BALB/c and C57BL/6 mice. (**A**): The expression patterns of 15 transcription factors in generating resident tissue/organ-specific identities of the representative tissue-resident Mø in the propagated lung tissue-resident Mø (plMø) and alveolar Mø (AMø). The expression patterns are almost the same in plMø and AMø between the two mouse types. The expression patterns are also similar between plMø and AMø, while that of *Pparg* is clearly expressed only in AMø, and that of *Smad3* and *Spic* are only expressed in plMø. (**B**): The expressions of *Csf1*, *Csf2* and *Il34* as well as *Tgfb1* in lung fibroblastic cells propagated by co-culturing with plMø in BALB/c and C57BL/6 mice. (**C**): The expressions of *Tgfb1* and *Tgfbr2* in plMø and AMø from BALB/c and C57BL/6 mice.

Past studies have shown that certain transcription factors ingenerate the resident tissue/organ-specific identities in several representative tissue-resident Mø [22,23]. Thus, we examined the mRNA expression of these transcription factors and a lineage-determining transcription factor of Mø, PU.1 as well, in propagated lung Mø by co-culture with lung fibroblastic cells, and compared their expression patterns between the propagated Mø and alveolar Mø. We found that (1) expression patterns of the transcription factors were quite similar between BALB/c and C57BL/6 mice (Figure 3A); (2) propagated lung Mø expressed *Bach1*, *Dtx4*, *Id2*, *Id3*, *Lxra*, *Smad2*, *Smad3*, *Spic* and *Pu.1* in both BALB/c and C57BL/6 mice, while the expressions of *Bach1,* *Smad3* and *Spic* were very low in the RT-PCR amplification of 32 cycles; (3) alveolar Mø expressed *Bach1*, *Dtx4*, *Id2*, *Id3*, *Lxra*, *Pparg*, *Smad2* and *Pu.1* in both BALB/c and C57BL/6 mice. These findings revealed that the expression patterns of the transcription factors were similar between lung Mø propagated by co-culture and alveolar Mø, while *Pparg* was clearly expressed only in alveolar Mø, and *Smad3* and *Spic* were expressed only in the propagated Mø.

### 3.4. Expression Profiles of Cytokines/Growth Factors in Lung Fibroblastic Cells and Tissue-Resident Mø propagated by Co-Culture as Well as Alveolar Mø

Tissue-resident Mø maintains their population through self-renewal under the tissue/organ-specific niche producing specific cytokines/growth factors, i.e., CSF-1, CSF-2 and IL34 [5,22,23]. Past reports have shown that CSF-2 and TGFβ1 are indispensable to self-maintain in alveolar Mø [9] and that TGFβ1 is essential for the maintenance of alveolar Mø and the regulation of alveolar Mø in an autocrine manner [26]. Thus, we examined the mRNA expression of these cytokines/growth factors in lung fibroblastic cells as well as that of TGFβ1 and TGFβR2 in propagated lung Mø and alveolar Mø. We found that the lung fibroblastic cells propagated by co-culture could clearly express *Csf-1*, *Il34* and *Tgfb1* mRNA in both BALB/c and C57BL/6 mice, but faintly expressed *Csf-2* mRNA additionally in BALB/c mice (Figure 3B); both the propagated lung Mø and alveolar Mø could clearly express *Tgfb1* and *Tgfbr2* mRNA in both BALB/c and C57BL/6 mice (Figure 3C). These findings demonstrate that the expression patterns of the cytokines/growth factors in lung fibroblastic cells were similar between the two mouse types, except for the *Csf-2* expression only in those of BALB/c; the expression patterns of the growth factor/growth factor receptor were similar between the propagated lung Mø and alveolar Mø and between the BALB/c and C57BL/6 mouse types.

### 3.5. Characterisation of Propagated Lung Tissue-Resident Mø by Flow Cytometry

**Figure 4:**
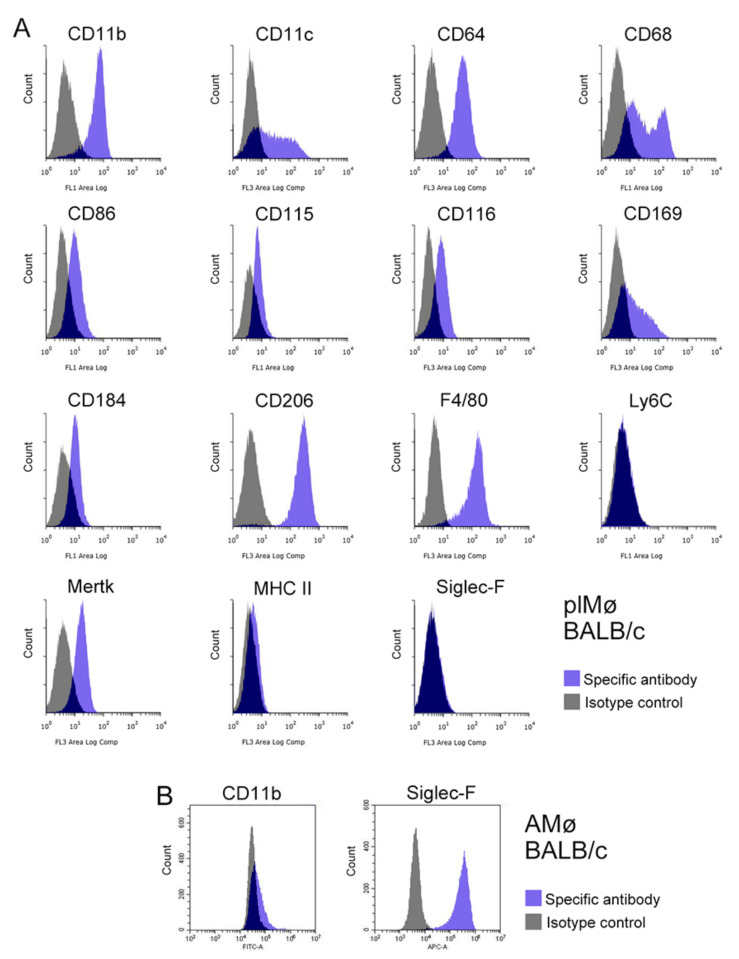
Representative histograms from flow cytometric analyses, showing the expression of 15 Mø/monocyte markers in propagated lung tissue-resident Mø by co-culturing as well as the CD11b and Siglec-F expression in alveolar Mø from BALB/c mice. (**A**): The expression patterns of CD11b, CD11c, CD64, CD68, CD86, CD115, CD116, CD169, CD184, CD206, F4/80, Ly6C, Mertk, MHC II and Siglec-F in propagated lung tissue-resident Mø (plMø) by co-culture and subculture (blue histogram, specific antibody; grey histogram, isotype control). Cell suspensions were pre-treated with an anti-mouse CD16/32 antibody and then treated with a fluorochrome-labelled test antibody or the same amount of fluorochrome-labelled isotype control antibody. plMø shows the characteristic expression patterns of Mø/monocyte marker membrane proteins, such as the high expressions of CD11b, CD64 and CD206; substantial/clear expressions of Mertk, CD115 and CD116; as well as no/almost no expressions of Ly6C, MHC II and Siglec-F. Low/faint and high expression fractions can be noted in CD11c, CD68 and CD169. (**B**): CD11b and Siglec-F expression in alveolar Mø (AMø) from BALB/c mice.

**Figure 5:**
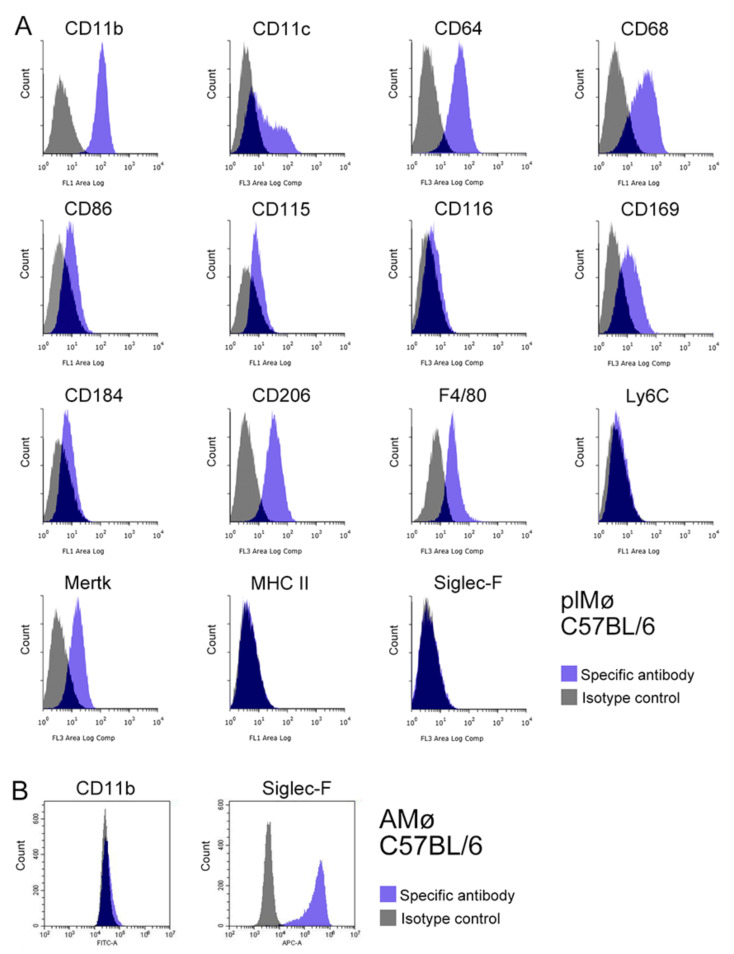
Representative histograms from flow cytometric analyses showing the expression of 15 Mø/monocyte markers in propagated lung tissue-resident Mø by co-culturing as well as CD11b and Siglec-F expression in alveolar Mø from C57BL/6/c mice. (**A**): The expression patterns of CD11b, CD11c, CD64, CD68, CD86, CD115, CD116, CD169, CD184, CD206, F4/80, Ly6C, Mertk, MHC II and Siglec-F in propagated lung tissue-resident Mø (plMø) in co-culture and by subculture (blue histogram, specific antibody; grey histogram, isotype control). plMø shows the characteristic expression patterns of Mø/monocyte marker membrane proteins, such as the high expressions of CD11b, CD64 and CD206; substantial/clear expressions of Mertk, CD115 and CD169; as well as no/almost no expressions of CD116, Ly6C, MHC II and Siglec-F. The low/faint and high expression fractions are present in CD11c. (**B**): CD11b and Siglec-F expression in alveolar Mø (AMø) from C57BL/6 mice.

The expression profiles of Mø markers (CD11b, CD11c, CD64, CD68, CD86, CD115, CD116, CD169, CD184, CD206, F4/80, Ly6C, Mertk, MHC II and Siglec-F) in propagated lung Mø segregated from subcultured lung fibroblastic cells obtained from BALB/c and C57BL/6 mice were examined by flow cytometry. Based on the histograms of the marker expression distribution, the propagated lung Mø largely showed similar expression profiles between BALB/c and C57BL/6. The propagated Mø from BALB/c mice revealed high expressions of CD11b, CD11c, CD64, CD68, CD206 and F4/80; substantial/clear expressions of CD86, CD115, CD116, CD169, CD184 and Mertk; and no/almost no expression of Ly6C, MHC II and Siglec-F, albeit only low/faint and high/clear expression fractions were noted in CD11c, CD68 and CD169 (Figure 4A). On the other hand, those from C57BL/6 mice revealed high expressions of CD11b, CD11c, CD64, CD68 and CD206; clear/substantial expressions of CD86, CD115, CD169, CD184, F4/80 and Mertk; and no/almost no expression of CD116, Ly6C, MHC II and Siglec-F, albeit only low/faint and high expression fractions were noted in CD11c (Figure 5A). These expression analyses clearly revealed that the propagated lung Mø segregated from the co-culture of lung fibroblastic cells were CD11b-highly-positive, CD64-highly-positive, CD206-highly-positive, Mertk-positive, MHC II-almost-negative, Ly6C-negative and Siglec-F-negative in both BALB/c and C57BL mouse types.

Then using alveolar Mø from the bronchoalveolar lavage as samples, we also examined the expressions of the marker molecules, CD11b, and Siglec-F that could clearly distinguish ex vivo lung interstitial Mø from alveolar Mø. Based on the histograms of CD11b and Siglec-F expression distribution, alveolar Mø from both BALB/c and C57BL/6 mice revealed faint/no expressions of CD11b and high expressions of Siglec-F (Figure 4B and Figure 5B). Thus, flow cytometry analyses suggested that the expression profiles of these molecules are clearly different between propagated lung Mø and alveolar Mø from both the BALB/c and C57BL mouse types.

### 3.6. M1/M2 Polarisation Induction of Propagated Mø by Co-Culturing with Lung Fibroblastic Cells

Mø derived from blood monocytes in BALB/c and C57BL/6 mice have been regarded as being more easily polarised to the M2 and M1 phenotypes, respectively, using polarisation reagents [19,20]. Thus, we further examined a polarisation property of propagated lung Mø from BALB/c and C57BL/6 mice to classical M1 and alternative M2 phenotype in response to stimulation with the combination of LPS/IFN-γ and IL-4, respectively. For this purpose, we examined the expression levels of iNOS and arginase 1 as a representative M1 and M2 polarisation marker, respectively, by flow cytometry.

**Figure 6:**
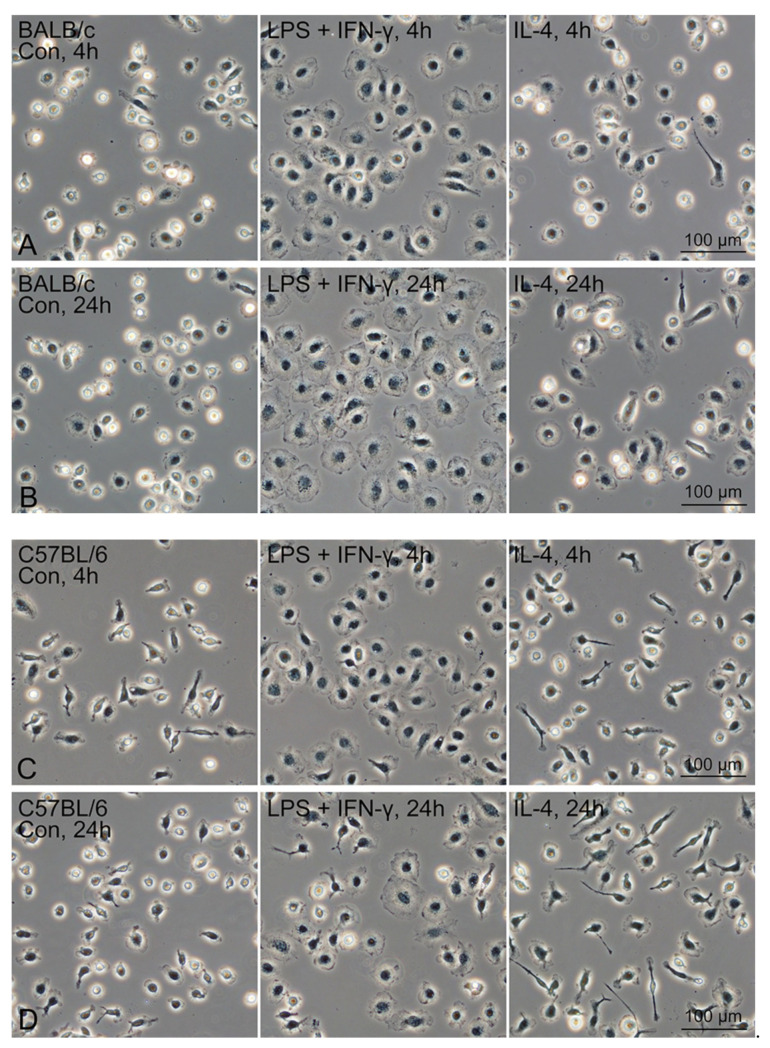
The morphology of propagated lung Mø treated with M1 (LPS plus IFN-γ) and M2 (IL-4) polarisation inducer for 4 h and 24 h. Representative phase-contrast images of BALB/c (**A**,**B**) and C57BL/6 (**C**,**D**) lung Mø treated with or without (Con) the inducers for 4 h (**A**,**C**) and 24 h (**B**,**D**). Flattened and round cells extending their cytoplasm frequently appeared at 4 and 24 h after the treatment with the LPS plus IFN-γ in both BALB/c and C57BL/6 mouse types.

Propagated Mø treated with the M1 and M2 polarisation inducers revealed similar morphological features between those from BALB/c and C57BL/6 mouse types. Flattened and round cells that extended their cytoplasm frequently appeared at 4 and 24 h after the treatment with the LPS plus IFN-γ in both BALB/c and C57BL/6 mice (Figure 6). In contrast, thin elongated cells (with a few long cytoplasmic protrusions) frequently appeared at 24 h after the treatment with IL-4 in C57BL/6 mice, while slightly extended round cells frequently appeared at 24 h after the treatment with IL-4 in C57BL/6 mice when compared with the morphology of the untreated control cells.

**Figure 7:**
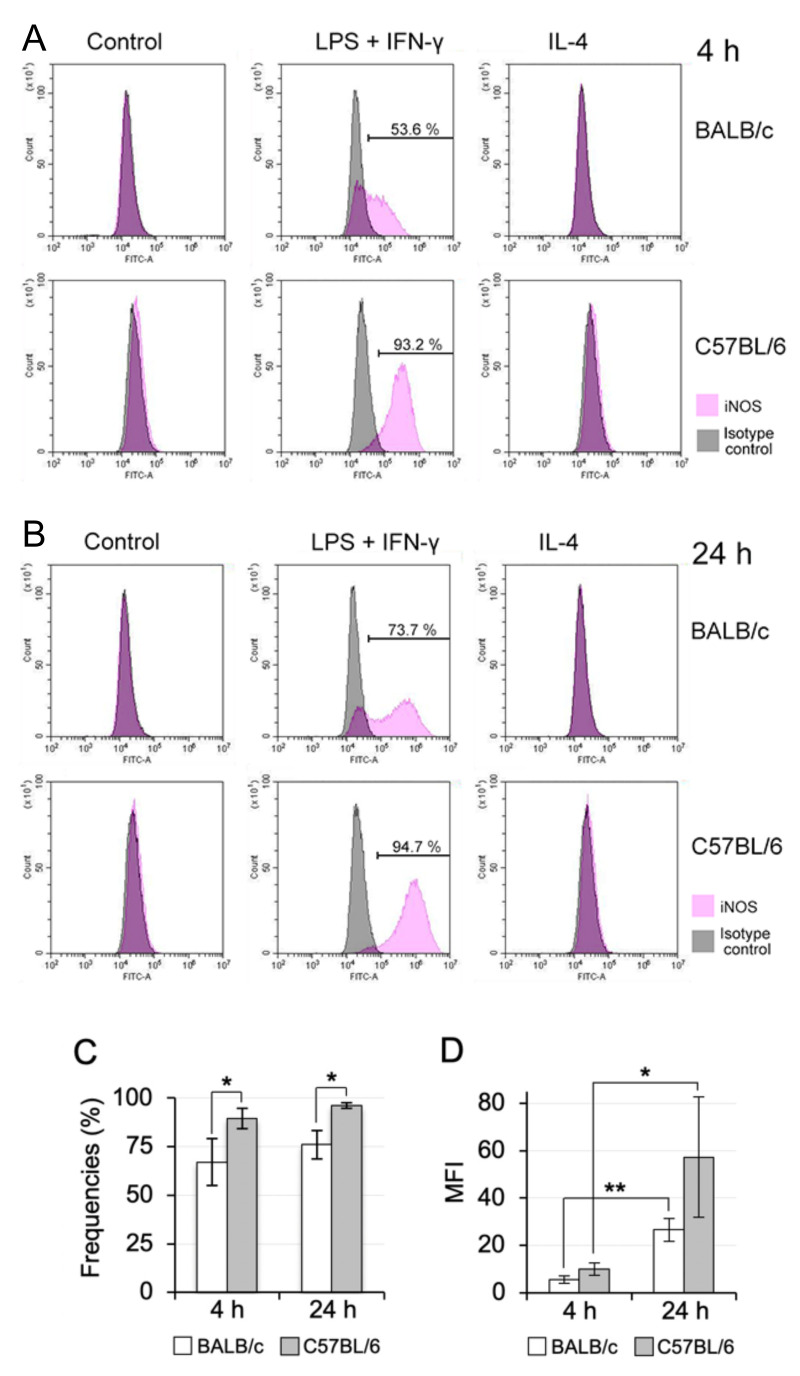
Comparison of the M1 polarisation property of BALB/c lung Mø propagated by co-culturing with that of C57BL/6 lung Mø. (**A**,**B**): Representative histograms from flow cytometric analyses demonstrating the expression of iNOS in propagated lung Mø of BALB/c mice and C57BL/6 mice treated with M1 (LPS plus IFN-γ) and M2 (IL-4) polarisation inducer or without inducers (Control) for 4 h (**A**) and 24 h (**B**). The iNOS expression was induced only in lung Mø of both the mouse types treated with the M1 inducer: lung Mø of BALB/c mice treated with the M1 inducer for 4 h and 24 h consisted of the iNOS-positive and -negative fraction, while those of C57BL/6 mostly consisted of the iNOS-positive fraction. (**C**,**D**): Frequencies showing iNOS-positive cells (**C**) and the mean fluorescence intensities (MFI) of iNOS-positive cells (**D**) in the lung Mø of BALB/c mice and C57BL/6 mice treated with LPS plus IFN-γ for 4 h and 24 h. Percentages and the MFI of iNOS-positive cells determined from three independent experiments of lung Mø from three mice and presented as mean ± SD (* *p* < 0.05, ** *p* < 0.01).

Based on the histograms of the marker expression distribution, flow cytometry revealed that the iNOS expression was clearly induced in the propagated Mø of both the BALB/c and C57BL/6 mouse types at 4 and 24 h after the treatment with LPS plus IFN-γ, while iNOS was not appreciably expressed in the control and treated cells with IL-4 (Figure 7A,B). Moreover, the iNOS expression induction in BALB/c Mø was weak at both the measurement time points relative to that in C57BL/6 Mø. In addition to an iNOS induction population, a non-induction population clearly appeared only in BALB/c lung Mø treated with LPS plus IFN-γ. Moreover, the expression levels of iNOS at 24 h after the treatment were high in both BALB/c and C57BL/6 lung Mø when compared with those at 4 h after the treatment. We quantitatively compared the percentages of iNOS-positive cells at both the measurement time points in lung Mø treated with the M1 polarisation induction reagents between BALB/c and C57BL/6 mice: iNOS-positive populations appeared at 67.1% ± 12.1% at 4 h and 76.0% ± 7.4% at 24 h in BALB/c lung Mø and 89.4% ± 5.3% at 4 h and 96.1% ± 1.3% at 24 h in C57BL/6 lung Mø; percentages of iNOS-positive cells were significantly different between BALB/c and C57BL/6 lung Mø at 4 h and 24 h after the treatment (Figure 7C; *p =* 0.029 at 4 h, *p =* 0.047 at 24 h). We also quantitated mean fluorescence intensities (MFI) of iNOS-positive cells of propagated lung Mø from BALB/c and C57BL/6 mice in the LPS plus IFN-γ-treatment group at both time points. The MIF appeared at 5.57 ± 1.54 at 4 h and 26.59 ± 4.80 at 24 h in BALB/c lung Mø, and the values were significantly different between the two time points (Figure 7D, *p =* 0.002). The MIF appeared at 10.06 ± 2.62 at 4 h and 57.17 ± 25.39 at 24 h in C57BL/6 lung Mø and the values were significantly different between the two time points (*p =* 0.033). The MIF values at 4 h and 24 h in C57BL/6 lung Mø tended to be higher compared with those at 4 h and 24 h of BALB/c lung Mø, respectively (*p =* 0.063 and *p =* 0.110).

**Figure 8:**
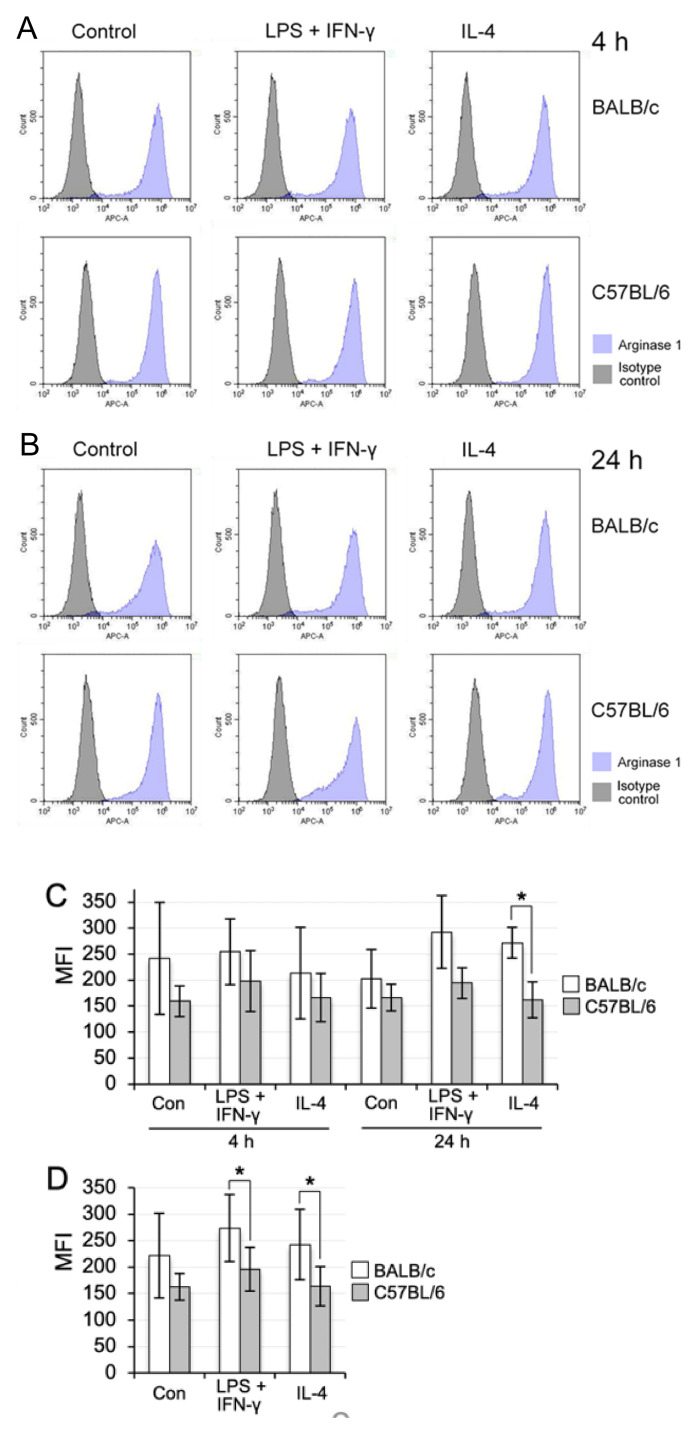
Comparison of the M2 polarisation property of BALB/c lung Mø propagated by co-culturing with that of C57BL/6 lung Mø. (**A**,**B**): Representative histograms from flow cytometric analyses showing the expression of arginase 1 in the propagated lung Mø of BALB/c mice and C57BL/6 mice treated with M1 (LPS plus IFN-γ) and M2 (IL-4) polarisation inducer or without inducers (Control) for 4 h (**A**) and 24 h (**B**). Arginase 1 was expressed in lung Mø with or without treatment of the M1 and M2 inducers. The expression levels were similar in BALB/c lung Mø among the three groups as well as in the C57BL/6 lung Mø among the three groups, while the arginase 1 expression levels were apparently high in the BALB/c lung Mø when compared with those in the C57BL/6 lung Mø in all three groups. (**C**,**D**): the mean fluorescence intensities (MFI) of arginase 1 expression of the untreated control (Con), LPS plus IFN-γ- and IL-4-treatment group in the propagated lung Mø of BALB/c and C57BL/6 mice at 4 h and 24 h (**C**) as well as those of the three groups without subdividing the two time points in each group (**D**). The MFI of arginase 1 expression determined from three independent experiments of lung Mø from three mice and presented as mean ± SD (*n* = 3 in C, *n* = 6 in D; * *p* < 0.05).

In contrast, arginase 1 was highly expressed in the propagated BALB/c and C57BL/6 lung Mø of all three groups: arginase 1 was highly expressed not only in the IL-4-treatment group but also in the LPS plus IFN-γ-treatment and control group at 4 h and 24 h after the treatments (Figure 8A,B). We compared the MFI of arginase 1 expression at both time points in propagated lung Mø of the control, LPS plus IFN-γ-, and IL-4-treatment group between BALB/c and C57BL/6 mice (Figure 8C). The MIF values at 4 h and 24 h in BALB/c lung Mø tended to be higher compared with those of C57BL/6 lung Mø, respectively, within each group, but there was no significant difference between the two mouse Mø within each group at the respective time points, except for the IL-4-treatment group at 24 h (*p =* 0.269 and *p =* 0.377 between the control groups at 4 h and 24 h, respectively; *p =* 0.319 and *p =* 0.089 between LPS plus IFN-γ-treatment groups at 4 h and 24 h, respectively; *p =* 0.462 and *p =* 0.014 between IL-4-treatment groups at 4 h and 24 h, respectively). We also compared the MFI values of arginase 1 in the control-, LPS + IFN-γ- and IL-4-treatment groups without subdividing the two time points in each group between BALB/c and C57BL/6 mice (Figure 8D). The MIF values of BALB/c lung Mø tended to be high compared with those of C57BL/6 lung Mø in the control (*p =* 0.115), whereas the MIF values were significantly different in the LPS + IFN-γ- and IL-4-treatment groups between BALB/c and C57BL/6 mice (*p =* 0.031 and *p =* 0.031, respectively).

## 4. Discussion

We successfully propagated tissue-resident Mø from the BALB/c and C57BL/6 mouse lung by co-culturing with lung fibroblastic cells, followed by subculturing in standard culture media containing 10% FBS without any additional growth factors. These Mø demonstrated high phagocytotic activities and the characteristic expression properties of Mø/monocyte marker membrane proteins, and the high expressions of CD11b, CD64 and CD206, and the substantial/clear expressions of Mertk as well as no expressions of Ly6C, MHC II and Siglec-F. These properties likely demonstrated that the propagated lung Mø is lung interstitial Mø of a certain population, and not alveolar Mø and lung monocytes based on the following three reasons: (i) alveolar Mø and monocytes present are in the alveolar lumen and the vascular lumen, respectively, in the mouse lung [12,15]. We used the lung intracardially perfused with HBSS including heparin to remove as much blood as possible to avoid contamination of the blood monocytes in the primary co-culture. Moreover, we removed the alveolar Mø from the lung through bronchoalveolar lavage before collecting cells for Mø propagation. Thus, the contamination of alveolar Mø and lung monocytes was possibly minimised in the collected cells for the primary co-culture. (ii) It is well accepted that alveolar Mø are CD11b-negative/-faintly positive and Siglec-F-highly positive cells and lung monocytes are CD206-negative and Mertk-negative cells [12]. These typical features of the characteristic marker expressions in alveolar Mø and lung monocytes do not match those of the propagated lung Mø. (iii) Recently, Shoyns et al. clearly demonstrated two functionally distinct populations of lung interstitial Mø in mice [15]. One interstitial Mø were CD206-negative and MHC II-positive cells exhibiting features of antigen-presenting cells that populate the alveolar interstitium and could possibly be gradually replaced by patrolling monocytes as putative precursors, and the other interstitial Mø were CD206-positive and MHC II-negative cells showing self-maintaining tissue-resident Mø that populate the peribronchial interstitium and constitutively produce high levels of chemokines and immunosuppressive cytokines. Thus, Mø maker expression features of the propagated tissue-resident Mø quite conform with those of the CD206-positive/MHC II-negative population of the lung interstitial Mø. Moreover, we demonstrated that the CD206-positive/MHC II-negative lung Mø propagates in vitro along with the propagation of lung fibroblastic cells. This finding may demonstrate a self-maintaining property of lung interstitial Mø of a certain population in vitro. The present findings may suggest that (1) the propagated lung Mø by co-culture possibly originates from foetal precursors because Tan et al. revealed two distinct populations of lung interstitial Mø in adult mice, one of which originated from the foetal precursors from the yolk sac [14]. Further studies by genetic lineage tracing on lung interstitial Mø are expected to definitely determine the origin of the propagated lung Mø.

We found that (1) the propagated lung Mø by co-culturing mostly contacted with lung fibroblastic cells; (2) the lung Mø after the isolation from the lung fibroblastic cells did not substantially propagate in the standard culture medium; (3) the lung Mø propagating in vitro substantially expressed CD115, the receptor for CSF1; (4) the lung fibroblastic cells from both BALB/c and C57BL/6 mice expressed *Csf1* clearly and *Il34* substantially, both of which induce the proliferation of several tissue-resident Mø [5,22,23]. These findings suggest that lung fibroblastic cells propagated by co-culture could act as the niche nursing lung interstitial Mø in vitro. If this presumption is correct, lung fibroblastic cells propagated by co-culturing may be a good tool to reveal the niche nursing lung interstitial Mø of the CD206-positive population. It is thus accepted that the development, maturation and self-maintenance of tissue-resident Mø derived from the foetal precursor are critically regulated by niche signals [22,23]; these niche signals in alveolar Mø are triggered by CSF2 as well as TGFß1, which act in an autocrine manner in alveolar Mø [9,26]. Moreover, niche-signal-dependent critical transcription factors have been determined in representative tissue-resident Mø [22,23]. Here, we demonstrated the *Tgfb1* expression in propagated lung Mø and fibroblastic cells as well as the *Tgfbr2* expression in propagated lung Mø. Our findings suggest that the TGF signal in an autocrine manner can possibly function in lung interstitial Mø, likely in alveolar Mø. Moreover, to the best of our knowledge, there are no studies showing a TFGß−TFGßR paracrine/autocrine signalling pathway between lung interstitial Mø and their niche-forming cells. Further studies are required to determine whether the TFGß1−TFGßR2 paracrine/autocrine signalling pathways shape the resident/specific properties of lung interstitial Mø. We also showed that the expression patterns of the transcription factors were quite similar between the propagated lung Mø and alveolar Mø, except for a few transcription factors, including *Pparg,* which critically characterises the properties of alveolar Mø [27], as was clearly expressed only in alveolar Mø. These observations also suggest a similarity between the two Mø, thus indicating that propagated lung Mø may be available as a substitute for alveolar Mø when PPARγ can be introduced in propagated lung Mø. Moreover, to our knowledge, there are no studies on niche-dependent transcription factors providing resident tissue-specific identities in lung interstitial Mø. Thus, further studies are required to determine which transcription factors expressed in propagated lung interstitial Mø or others shape the resident/specific properties of lung interstitial Mø.

It has been reported that Mø derived from blood monocytes in BALB/c and C57BL/6 mice were more easily polarised to the M2 and M1 phenotypes, respectively [19,20]. A recent quantitative PCR study showed (1) high expression of *Nos2* and no or very low expression of *Ym1* as a M2 marker in one-day cultured ex vivo C57BL/6 mouse lung interstitial Mø separated as CD11b-positive cells and (2) up-regulation of *Nos2* and *Ym1* in interstitial Mø treated with 10 ng/mL IFN-γ and 10 ng/mL IL-4, respectively, for 24 h [28]. This indicates that ex vivo mouse lung interstitial Mø are likely polarised to the M1 phenotype. In the present study, we examined whether tissue-resident Mø are similarly polarised into M1/M2 phenotypes using propagated lung Mø from BALB/c and C57BL/6 mice through flow cytometry. We found that the lung Mø of both the mouse types expressed arginase 1 with or without treatment of the M1 and M2 polarisation inducers. This finding indicates that the propagated tissue-resident Mø were intrinsically polarised to the M2 phenotype in the co-culture with lung fibroblastic cells because arginase 1 is one of the most representative and reliable M2 Mø markers [20]. Moreover, this finding is not consistent with the previous study showing the M1 state of ex vivo lung interstitial Mø. We also found that the propagated lung Mø definitely expressed CD206 and *Tgfb1*, both of which are a representative M2 Mø marker and an inducer to elevate the M2 response, respectively [20]. Moreover, it has been reported that ex vivo lung interstitial Mø showing a self-maintaining property can characteristically express CD206. Thus, the propagated Mø by co-culturing with lung fibroblastic cells could possibly maintain the Mø polarisation properties in vivo. Further studies are warranted to examine this assumption. However, in contrast, we found that the M1 inducers could significantly increase the frequencies of iNOS-positive cells in C57BL/6 mice when compared to that in BALB/c mice. This difference may indicate that the lung tissue-resident Mø in the mouse strains possessed a similar M1 polarisation property to that of blood monocyte-derived Mø (recruited Mø), although the propagated lung Mø were already polarised to M2 in the steady-state condition in vitro.

This is the first study to demonstrate the essential properties of lung tissue-resident Mø propagated by co-culturing with lung fibroblastic cells. The propagated Mø were possibly identified as lung interstitial Mø based on the features of CD206- and CD11b-highly positive, MHC II-negative and Siglec-F-negative cells as well as the *Tgfb1*- and *Tgfbr2*-expressing cells. Severe acute respiratory syndrome−coronavirus 2 (SARS-CoV-2) infection results in a hyperinflammatory syndrome, which has been reported as the major cause of death, at a low frequency and bilateral interstitial pneumonia, often leading to acute respiratory distress syndrome and pulmonary fibrosis at significant frequencies. It has been reported that the dysregulated responses of alveolar Mø and recruited Mø are deeply involved in the pathology of the hyperinflammatory syndrome and pulmonary fibrosis [16,18]. Lung interstitial Mø, which is another population of tissue-resident Mø residing in the lung. However, not much attention has been paid to their involvement in the pathologies caused by SARS-CoV-2 infection. Therefore, propagated tissue-resident lung Mø by co-culturing seems to be an indispensable cell tool for investigating the pathology and drug development of SARS-CoV-2 disease, especially for pulmonary fibrosis due to their M2 polarisation properties. Moreover, we found that the propagated tissue-resident Mø expressed CD169 (Siglec-1, sialic acid-binding immunoglobulin-like lectin 1; Sialoadhesin), which can bind with N-acetylneuraminic acid (Neu5Ac)—the most abundant mammalian sialic acid—and mediate trans-infection of murine leukaemia virus in a Neu5Ac side chain-dependent manner [29,30]. Accumulating evidence suggests that the N-terminal domain of SARS-CoV-2 spike protein can bind to Neu5Ac on the cell surface of host cells, while the C-terminal domain can bind to angiotensin-converting enzyme receptor 2 (ACE2) [31]. Thus, the lung tissue-resident Mø expressing CD169 may interfere with SARS-CoV-2-binding to ACE2-expressing host cells. Therefore, the propagated lung Mø may act as a useful cell tool to investigate SARS-CoV-2 infection. We expect that the lung tissue-resident Mø possesses a high ability of propagation when co-cultured with lung fibroblastic cells, demonstrating features of lung interstitial Mø for use on diverse applications in lung diseases.

## 5. Conclusions

**Figure 9:**
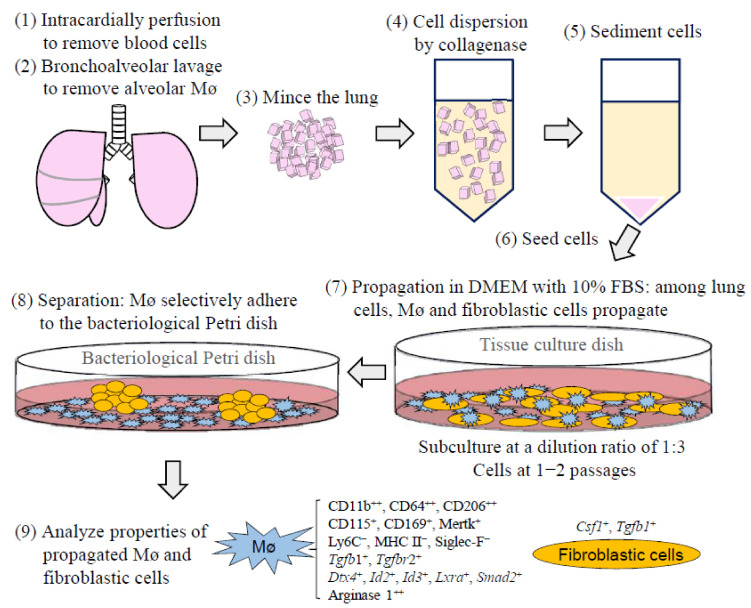
Schematic drawing illustrating the procedures of lung tissue-resident Mø propagation by co-culture with lung fibroblastic cells and separation of lung Mø from fibroblastic cells as well as the expression characteristics of these cells.

Lung tissue-resident Mø of BALB/c and C57BL/6 mice could be propagated in large numbers under standard culture media along with the propagation of lung stromal cells demonstrating fibroblastic morphology (Figure 9). The propagated lung Mø by co-culturing demonstrated characteristic expression patterns for Mø/monocyte markers: high expressions of CD11b, CD64 and CD206; substantial expressions of Mertk; and negative expressions of Ly6C, MHC II and Siglec-F. These expression properties quite fit with those of lung interstitial Mø of a certain population with the property of self-renewal. The propagated lung Mø from both the mouse types were polarised to an M2 phenotype that highly expressed arginase 1 without treatment of the M2 inducer, whereas the M1 inducers could significantly increase the percentages of iNOS-positive cells in C57BL/6 mice when compared to those in BALB/c mice. Moreover, the propagated fibroblastic cells by co-culturing with lung tissue-resident Mø possessed the properties of niche such as the expressions of *Csf1* and *Tgfb1*. This is the first study to demonstrate the fundamental properties of lung tissue-resident Mø propagated by co-culturing. We, therefore, expect that the lung tissue-resident Mø possesses a high ability of propagation when co-cultured with lung fibroblastic cells, demonstrating features that are almost identical to those of lung interstitial Mø for use in diverse applications for lung diseases, including SARS-CoV-2 disease.

## Data Availability

The data presented in this study are available on request from the corresponding author.

## Supplementary Materials

The following are available online at https://www.mdpi.com/article/10.3390/biomedicines9091241/s1. Table S1, Primers of transcription factors and cycle numbers for PCR amplification; Table S2, Primers of factors proliferating/characterising macrophages and cycle numbers for PCR amplification; Table S3, Monoclonal antibodies used for flow cytometry.
